# Clinical study of ultrasound-guided transversus abdominis plane block for analgesia after cesarean section

**DOI:** 10.1097/MD.0000000000017542

**Published:** 2019-10-11

**Authors:** Yuanchao Gao, Mengzhuo Guo, Chunyan Du, Haijing Zhang, Huan Zhang

**Affiliations:** Department of Anesthesiology, Beijing Tsinghua Chang Gung Hospital, Changping District, Beijing, China.

**Keywords:** analgesia, cesarean section, PCIA, TAP, ultrasound-guided

## Abstract

**Background:**

Patient-controlled intravenous analgesia (PCIA) and patient-controlled epidural analgesia are 2 common methods of maintaining analgesia after cesarean section. In recent years, transversus abdominis plane block (TAPB) has been gradually applied clinically to reduce opioid analgesics and has achieved good results. Therefore, we performed this study to compare the efficacy and side effects of TAPB and PCIA in analgesia after cesarean section.

**Methods:**

One hundred patients who underwent cesarean section were randomly classified into 2 groups. Following surgery, one group underwent ultrasound-guided TAPB and the other group underwent PCIA. Pain intensity according to the visual analog scale (VAS; 0 for no pain and 10 for severe intolerable pain) was assessed at 2, 4, 6, 8, 12, and 24-hour postsurgery in both groups. The postoperative complication rate and patient satisfaction were also measured.

**Results:**

No significant differences were found in the VAS scores between the groups (*P* > .05). However, the incidence of postoperative complications in the TAPB group was significantly lower than that in the PCIA group (*P* < .05). Furthermore, patient satisfaction in the TAPB group was significantly higher than that in the PCIA group (*P* < .05).

**Conclusion:**

This study demonstrated that ultrasound-guided TAPB can achieve the same analgesic effect as PCIA after cesarean section but with even higher patient satisfaction.

## 1 Introrduction

With the enactment of the 2nd birth policy in China, an increasing number of people want a 2nd child. The new policy has also resulted in an increased number of cesarean sections being performed. Thus, postpartum analgesia has become a common concern. There are 2 traditional analgesic techniques: patient-controlled intravenous analgesia (PCIA) and patient-controlled epidural analgesia. Conventionally, opioids are the drugs that are most commonly used to provide effective pain relief during the postoperative period. However, opioids are associated with dose-dependent side effects, such as nausea, vomiting, pruritus, sedation, and respiratory depression,^[1]^ which complicate perioperative care. The management of postoperative pain in these patients is equally challenging. Using pain control approaches without using opioids can effectively improve the quality of postoperative recovery. Presently, the most popular regional block after cesarean section is transversus abdominis plane block (TAPB).^[2–4]^ TAPB is an effective technique to counter postoperative pain after abdominal surgery, particularly in patients undergoing postcesarean section.^[5]^ Ultrasound-guided TAPB could vastly improve the success of inguinal nerve blocks, reduce the volume of local anesthetic used, and prevent potential injury of adjacent structures.^[6]^ This study was performed to compare PCIA and TAPB in sparing postoperative rescue analgesics under spinal anesthesia and to provide rational coping strategies.

## 2 Methods

Using a computer-generated random table, we selected 103 patients at the Beijing Tsinghua Changgung Hospital with American Society of Anesthesiologists class I and II physical status who were aged 26 to 40 years and were planned for emergency or elective lower segment cesarean section with spinal anesthesia. The study protocol and advantages and potential disadvantages of the block methods were explained to the participants, and those who provided written consent were included in the study. The exclusion criteria included lack of written consent, drug abuse, allergic reactions to local anesthetics, body mass index >35, body weight <60 kg, coagulopathies, and requirement of general anesthesia. In the operating room, an intravenous cannula (18-gage) was inserted into the hand or arm of all patients. The patients were then monitored by noninvasive blood pressure, electrocardiogram, and pulse oxygen saturation; fluid therapy was given using 500 cc of Ringer lactate solution. The patients were turned to the left lateral position and were administered single-injection spinal anesthesia under sterile conditions using a 25-G needle in the L3–L4 space with 2.5 mL of 0.5% ropivacaine. Surgery was initiated after attaining T8 sensor blockade, and each case was managed by the same anesthesiologist without using any narcotics intraoperatively.

Following surgery, the patients were randomly divided into 2 groups: TAPB group (T group; n = 51) and PCIA group (P group; n = 52). In the TAPB group, the probe was placed perpendicular to the mid-axillary line between the iliac crest and subcostal margin, the 3 abdominal muscle layers were identified, and the transversus abdominis plane was located between the internal oblique and transversus abdominis muscle. Next, 30 mL of 0.33% ropivacaine was injected, and drug spread in the plane was observed. The same procedure was repeated on the other side of the abdomen. In the PCIA group, following surgery, the patients were administered PCIA comprising sufentanil 100 mcg and 98 mL of 0.9% physiological saline in their intravenous cannula at a background dose of 2 mL/hour.

The pain intensity according to the visual analog scale (VAS; 0 for no pain and 10 for severe intolerable pain) was compared at 2, 4, 6, 8, 12, and 24 hours postoperatively in both groups to evaluate the effectiveness in relieving pain, and side effects such as nausea, vomiting, pruritus, sedation, and respiratory depression were recorded. Furthermore, if the patient complained of pain (VAS > 4), they were provided language comfort and were told that lactation was required the following day and analgesics may be secreted through milk. The patients stated that they understood and could endure the pain.

The analgesic satisfaction 24 hours after surgery was measured as follows: 0 = weak, 1 = medium, 2 = good, 3 = very good, and 4 = excellent. The recovery time of intestinal peristalsis (anal exhaust time) after surgery and time required for the patient's first ambulation were also recorded.

### 2.1 Statistical analysis

We recruited at least 50 patients in each group to minimize any impact of data loss. SPSS 18 was used to statistically analyze the collected data, with a 2-tailed *P* value of <.05 considered significant. The Student *t* test was used for parametric data, and the Chi-squared test was used to compare differences between the variables obtained.

## 3 Results

A total of 103 patients were enrolled in the study. Three patients (1 in the TAPB group and 2 in the PCIA group) were excluded because of the loss of postoperative follow-up records. Thus, the results for 100 patients were analyzed in total. The general characteristics (age, height, body mass, American Society of Anesthesiologists classification, and operation time) between the 2 patients groups were not statistically significant (*P* > .05) (Table 1).

**Table 1:**
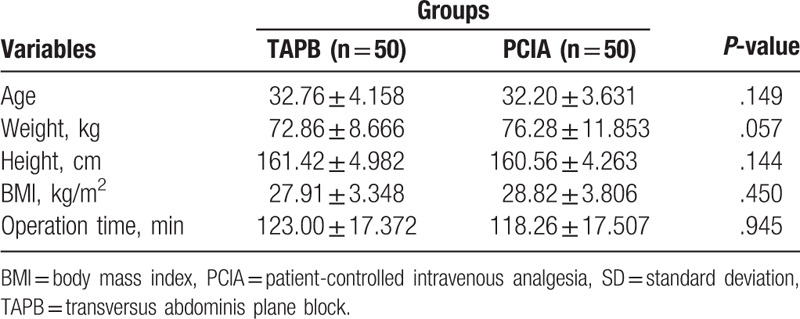
Patient physical characteristics and procedural data (mean ± SD).

No significant difference was found in the VAS scores at all time intervals after surgery between the groups. Some of the pregnant women (3 in the PCIA group and 5 in the TAPB group) required verbal comfort; however, they reported that they could tolerate the pain and required no analgesics (Table 2). In addition to one case of nausea in the TAPB group, no other related complications, such as vomiting, dizziness, pruritus, sedation, and respiratory depression, occurred during the first 24-hour study period. However, in the PCIA group, 8 patients became nauseated, 1 patient was slightly sedated, and 4 patients vomited. A statistically significant difference was found between the study groups. However, no pruritus and respiratory depression occurred in both groups (Table 3). Patient satisfaction was significantly higher in the TAPB group than in the PCIA group (Table 4).

**Table 2:**
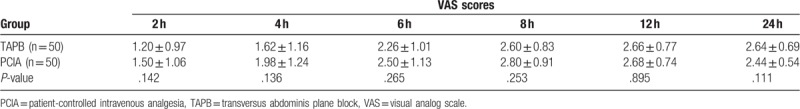
Comparison of 24-h VAS scores between the groups.

**Table 3:**
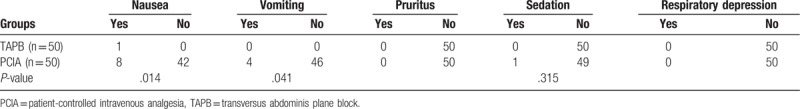
Comparison of postoperative complications between the groups.

**Table 4:**
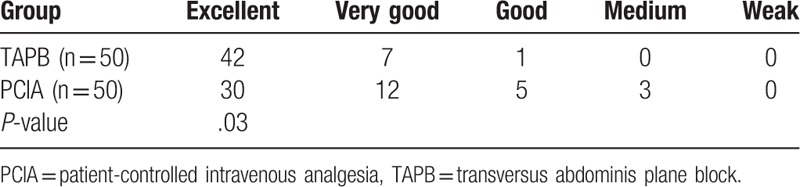
Comparison of the overall satisfaction of postoperative analgesia between the groups.

## 4 Discussion

Provision of analgesia or anesthesia to the anterior abdominal wall by TAPB has been well documented. The technique yields high analgesic efficacy and potentially long duration, with reported analgesia lasting up to 36 hours.^[7]^ TAPB is associated with lower incidences of hypotension and motor blockade.^[7]^ According to previous study results, the use of local anesthesia reduces the need for analgesic medications and reduces pain after surgery.^[8]^ Using TAPB to reduce pain after cesarean section and hysterectomy improved patient recovery.^[7,9]^ Under ultrasound guidance, the accuracy of TAPB is markedly improved. According to the results of this study, ultrasound-guided TAPB has the same analgesic effect as traditional PCIA on pain control after cesarean section, but TAPB provides higher patient satisfaction because of its convenience, lack of pump requirement, and lack of postoperative complications. Additionally, the minor effect on free movements in the TAPB groups resulted in added comfort. However, systemic and intrathecal usage of opioids after cesarean section results in obvious side effects and limits, such as respiratory depression, nausea, vomiting, itching, excessive sedation, reduced peristaltic intestine activity, and pruritus. These side effects make patients uncomfortable and might affect the prognosis.

Numerous authors believe that the most effective and longest-lasting analgesic duration is 11 to 24 hours in the postoperative period among patients after undergoing cesarean section.^[10,11]^ This study focused on assessing pain in the postoperative period during the first 24 hours after cesarean section. On the 2nd day following surgery, the pain was not very severe and was markedly relieved. Even a very small amount of drug without analgesics can achieve a good analgesic effect. TAPB markedly avoids the influence of drug secretion with milk, vastly benefiting infants.

Various analyses have shown that TAPB is an effective tool to counter postoperative pain in terms of reducing the total dose of opioids but only when spinal morphine is not used.^[12]^ In the present study, TAPB was performed after surgery and morphine and other opioids were not used outside the epidural space. A previous study^[13]^ demonstrated that ultrasound-guided TAPB using 20 cc of ropivacaine 0.2% was effective on both sides for postoperative pain management in patients who had undergone cesarean section. However, in addition to lateral abdominal block, 1 g of Apotel was injected intravenously every 8 hours within 15 minutes, and 25 mg of meperidine was injected intravenously for patients with a VAS score of >3. Therefore, in this study, 30 mL of 0.3% ropivacaine was administered to both sides, thereby not only achieving the analgesic effect but also prolonging the analgesic time. The maximum amount of ropivacaine was not exceeded. Another study^[14]^ reported that TAPB was performed by utilizing anatomical landmarks for better analgesia in lower parts of the abdomen; however, using this block method has a higher risk of complications such as nerve and organ damage.

Because of the use of ultrasound in blocks, truncal blocks can be simply performed with a low frequency of side effects.^[15]^ In this study, we also conducted bilateral TAPB under ultrasound guidance for patients undergoing cesarean section without complications and with definite effects. No significant difference was found between the groups in pain scores recorded at regular time intervals both at rest and during movement. Few side effects were noted in the TAPB group, whereas 1 patient vomited, 1 was sedated slightly, and 3 were nauseated in the PCIA group. Patient satisfaction in the TAPB group was significantly higher than that in the PCIA group, likely resulting from more effective pain control by TAPB after cesarean section.^[13,16]^ In addition, TAPB was better accepted because it was more convenient for patients.

Overall, the present study showed that compared with traditional PCIA, ultrasound-guided TAPB, as a local analgesic mode, exhibits fewer side effects and threats to patient health and vastly improves patient satisfaction. It is a better strategy for pain control after cesarean section in clinical practice, especially in pregnant woman, and greatly improves the quality of life.

## Author contributions

**Conceptualization:** Yuanchao Gao, Huan Zhang.

**Data curation:** Yuanchao Gao, Mengzhuo Guo, Chunyuan Du, Haijing Zhang.

**Formal analysis:** Yuanchao Gao, Mengzhuo Guo, Chunyuan Du.

**Investigation:** Yuanchao Gao, Chunyuan Du.

**Writing – original draft:** Yuanchao Gao.

**Writing – review & editing:** Huan Zhang.
